# Research hotspots and trends in electrical stimulation for movement disorders: a bibliometric analysis from 2016 to 2025

**DOI:** 10.3389/fnagi.2026.1793056

**Published:** 2026-04-23

**Authors:** Anqi Liu, Zixuan Shu, Jingqi Wen, Xinyu Cui, Yi Shi, Shaohui Geng

**Affiliations:** 1 Department of Dermatology, Shenzhen People’s Hospital (The First Affiliated Hospital, Southern University of Science and Technology, The Second Clinical Medical College, Jinan University), Shenzhen, China; 2 School of Chinese Pharmacy, Beijing University of Chinese Medicine, Beijing, China; 3 Second Affiliated Hospital, Beijing University of Chinese Medicine, Beijing, China; 4 School of Life Science, Beijing University of Chinese Medicine, Beijing, China

**Keywords:** bibliometric analysis, CiteSpace, electrical stimulation, movement disorders, research trends, VOSviewer

## Abstract

Electrical stimulation therapies have become pivotal interventions for managing refractory movement disorders. However, the rapidly expanding and fragmented body of literature necessitates a macroscopic evaluation to identify global collaborative networks and emerging scientific frontiers. Consequently, this study conducted a comprehensive visualization analysis of research trends and hotspots in this domain to delineate the evolutionary trajectory and guide future therapeutic developments. Publications from 2016 to 2025 were retrieved from the Web of Science Core Collection and PubMed using keywords such as movement disorders, deep brain stimulation, and neuromodulation. Selection criteria mandated English-language clinical articles and reviews investigating electrical stimulation, while excluding purely pharmacological studies. Bibliometric mapping was performed utilizing CiteSpace VOSviewer, the bibliometrix R package, and Microsoft Excel. A total of 2,944 publications were retrieved from 553 journals by 13,489 authors from 489 countries/regions across six continents. The United States contributed the highest volume, followed by Germany and China. The University of Toronto emerged as the most prolific and collaborative institution ahead of Harvard Medical School and the University of Florida. The journal Movement Disorders ranked first in both publication productivity and co-citation frequency. Furthermore, Lozano AM and Fasano A were identified as the most productive authors, while Deuschl G and Benabid AL represented the foundational co-cited authorities. Thematic analysis indicates that research focuses primarily on deep-brain stimulation targeting the subthalamic nucleus for Parkinson’s disease. Crucially, emerging keywords, including plasticity, substantia nigra balance, and dynamics, demonstrate a definitive scientific shift. This transition moves away from static symptom management toward investigating complex neural circuit mechanisms and optimizing adaptive closed-loop neuromodulation to address refractory symptoms. This analysis highlights a major shift in electrical stimulation research from open-loop validation toward individualized connectomics and mechanism-based neurorehabilitation. Despite robust growth in publications, structural isolation persists in emerging high-productivity regions, necessitating multinational collaboration for future trials. Ultimately, harnessing targeted neuroplasticity and adaptive technologies will drive sustained functional neurorestoration and improve long-term patient outcomes.

## 1 Introduction

Motor disorders, including Parkinson’s disease, dystonia, tremor, and tic disorders, impose an escalating socioeconomic and healthcare burden globally, particularly in the context of an aging population (Espay et al., 2018). While pharmacological interventions remain the first-line treatment for many of these conditions, long-term use often leads to diminished efficacy, drug resistance, and severe adverse effects (Alharbi et al., 2024; Olanow et al., 2001). These clinical limitations highlight an urgent need to develop non-pharmacological neuromodulation strategies that can provide sustained symptom relief and promote functional neuroplasticity. Consequently, electrical stimulation therapy has emerged as a pivotal research frontier in neuroscience and rehabilitation medicine.

To address this clinical demand, various electrical stimulation modalities have been developed, spanning from mechanistic exploration to clinical applications. Invasive techniques represented by deep brain stimulation (DBS) and spinal cord stimulation (SCS) enable direct modulation of targeted neural circuits, significantly improving motor fluctuations in Parkinson’s disease (Limousin and Foltynie, 2019) and restoring neurological function in conditions like spinal muscular atrophy (SMA) (Prat-Ortega et al., 2025). Concurrently, non-invasive brain stimulation (NIBS) techniques, particularly transcranial direct current stimulation (tDCS) and transcranial magnetic stimulation (TMS), have been widely validated for their efficacy in enhancing post-stroke motor recovery and alleviating symptoms by modulating inherent nervous system networks (Lee et al., 2025; Tscherpel et al., 2020; Qin et al., 2025; Shi et al., 2025). Recent evidence highlights its profound capacity to activate specific brain regions through ascending neural pathways (Guo et al., 2024) and facilitate structural neuroplasticity for motor rehabilitation, particularly in post-stroke recovery (Zhu et al., 2024). Beyond central nervous system targets, peripheral electrical neuromodulation, such as transcutaneous electrical acupoint stimulation (TEAS) and electroacupuncture, has demonstrated significant potential in mitigating muscle atrophy during structural rehabilitation (Xue et al., 2025) and promoting microenvironmental tissue repair in spinal cord injuries (Kaitan et al., 2022).

The rapid growth of research on electrical stimulation for movement disorders has generated a substantial yet fragmented body of literature within clinical neurology and rehabilitation medicine. Although traditional reviews provide rigorous assessments of targeted therapies, their inherent focus on specific clinical outcomes limits their ability to reveal global research networks and emerging frontiers. Bibliometrics, as a robust quantitative analysis tool integrating mathematical and statistical methods, serves as a crucial complementary approach to overcome these analytical limitations (Olanow et al., 2001). By objectively visualizing the knowledge structure, bibliometric analysis effectively maps the evolutionary trajectory of the field, which is crucial for identifying critical knowledge gaps, tracking emerging therapeutic hotspots, and guiding future research directions (Aria and Cuccurullo, 2017).

Consequently, this study employs CiteSpace, VOSviewer, and the bibliometrix R package to conduct a comprehensive visual analysis of the literature on electrical stimulation for movement disorders published between 2016 and 2025. To ensure maximum data sensitivity and minimize retrieval bias, our search strategy was designed to encompass major neuromodulation modalities mentioned above. We primarily aim to delineate temporal trends in publication and map global collaborative networks among leading countries, institutions, and authors. Furthermore, we seek to identify the core journals and foundational literature constructing the knowledge base of this domain. This study also intends to trace the evolutionary trajectory of research hotspots and elucidate emerging frontiers, such as the transition toward non-invasive stimulation techniques and specific mechanistic investigations. By fulfilling these objectives, we endeavor to draw definitive conclusions regarding the current developmental stage and future directions of electrical stimulation therapies in movement disorders.

## 2 Methods

### 2.1 Search strategy

To ensure a comprehensive and reproducible literature retrieval, we developed and executed systematic search strategies in two major bibliographic databases: the Web of Science Core Collection (WoSCC) and PubMed.

For WoSCC, the search strategy relied on an extensive combination of free-text terms. These terms were initially identified through a preliminary search in PubMed to explore MeSH terms related to movement disorders and electric stimulation therapy, from which core concepts and their subordinate terms were derived, and supplemented with relevant free-text terms in the field. The final search query for WoSCC was: (TS = (Movement Disorder*) OR TS = (Dyskines*) OR TS = (Etat Marbre) OR TS = (Status Marmoratus) OR TS = (Atax*)) AND (TS = (Electric* Stimulation Therapy) OR TS = (deep brain stimulation) OR TS = (spinal cord stimulation) OR TS = (Therapeutic Electric Stimulation) OR TS = (TMS) OR TS = (tDCS) OR TS = (neuromodulation)).

To obtain search results comparable to those from WoSCC in PubMed, we designed a complementary search strategy combining MeSH terms with free-text terms in titles and abstracts: (“Movement Disorders”[Mesh] OR movement disorder*[Title/Abstract] OR dyskines*[Title/Abstract] OR atax*[Title/Abstract]) AND (“Electric Stimulation Therapy”[Mesh] OR electric* stimulation[Title/Abstract] OR deep brain stimulation[Title/Abstract] OR TMS[Title/Abstract] OR spinal cord stimulation[Title/Abstract] OR tDCS[Title/Abstract] OR neuromodulation[Title/Abstract]).

To ensure consistency across databases, the search in PubMed was limited to the 2004 to 2025 period based on the earliest available publications in the Web of Science Core Collection in 2004. To prioritize the investigation of contemporary developmental trends, we specifically selected the most recent ten-year literature from 2016 to 2025, a period where research progress updates and iterates rapidly. This deliberate analytical decision, combined with the availability of the full historical dataset in Supplementary Table S1, ensures methodological transparency and perfectly aligns with our core research aims.

### 2.2 Literature screening

The literature screening process was conducted based on predefined inclusion and exclusion criteria.

For WoSCC, records were initially retrieved and then screened by publication type, with non-article and non-review records excluded. Subsequently, studies published outside the 2016–2025 timeframe or categorized as Early Access were removed. Non-English publications were then excluded.

For PubMed, records were initially retrieved and then filtered by publication type, retaining clinical trials (all phases and designs), introductory articles, and reviews (including meta-analyses, systematic reviews, and scoping reviews). The search was then restricted to studies published within the 2016–2025 timeframe, followed by the exclusion of non-English publications.

Following the merging of records from both databases, duplicate entries were removed using CiteSpace’s built-in deduplication function. The remaining records underwent manual screening of titles, abstracts, and keywords based on predefined exclusion criteria: studies that did not employ electrical stimulation as the primary intervention (e.g., studies focusing solely on pharmacological interventions such as botulinum toxin), or that addressed conditions not manifesting as movement disorders (e.g., insomnia, obsessive-compulsive disorder, depression). Additionally, to ensure the quality and academic impact of the included literature, studies published in journals not indexed in the Science Citation Index Expanded (SCIE) or the Social Sciences Citation Index (SSCI) were excluded. Through this multi-step screening process, the final set of records was obtained for subsequent bibliometric analysis.

### 2.3 Data analysis

This study utilized CiteSpace (version 6.3. R1), VOSviewer (version 1.6.19), R packages, and Microsoft Excel 2021 for bibliometric analysis and visualization (Ding and Yang, 2022). CiteSpace was employed to analyze collaboration relationships among countries and authors, co-citation relationships of authors, keyword co-occurrence relationships, clustering, and timeline analysis. VOSviewer is a free Java-based software developed by Dutch scholars Van Eck and Waltman in 2009 (Van Eck and Waltman, 2010). In this study, this tool was used for visualizing keywords, countries, institutions, and author collaborations among scientific journals, as well as for visualizing cited journals and density visualizations of co-cited authors, cited journals, and document coupling. Additionally, the bibliometrix analysis webpage, which had been set up to operate within the R language environment, was used to display country association maps and analyze author publication timelines (Donthu et al., 2021). Excel software was also employed to analyze annual publications. To ensure data quality before formal analysis, all retrieved bibliometric records underwent preprocessing, which involved manually standardizing author names and institutional affiliations to resolve inconsistencies. Document and citation thresholds were determined based on data distribution patterns to balance meaningful representation with sufficient node inclusion, thereby ensuring the robustness of the network analysis. Detailed configuration parameters for each tool are specified in the corresponding methodological sections.

#### 2.3.1 Annual publication trend analysis

Microsoft Excel 2021 was used to analyze the annual number of publications related to the topic. An exponential trend line was fitted, and the R² value was calculated to assess the growth pattern.

#### 2.3.2 Country and institution collaboration analysis

To examine the geographical distribution and collaborative patterns in this research field, a combination of country and institution collaboration analyses was employed. This approach aims to reveal which nations are leading the research effort and how they collaborate internationally, providing insights into global research priorities and partnerships. Institution-level analysis identifies key research hubs and their collaborative networks, which are essential for understanding the organizational landscape and potential centers of excellence. Clustering analysis was applied to identify natural collaborative groupings and research communities.

CiteSpace was configured to analyze collaboration relationships among countries, with the node type set to “country” and the time span limited to 2016–2025 with one-year slices. The selection criteria were set to the top 15 most cited or productive countries per slice. The resulting network visually displayed the leading countries in publications related to electric stimulation therapy for movement disorders, highlighting their collaborative relationships. VOSviewer was used to construct a country collaboration network based on co-authorship data. The analysis type was set to co-authorship with countries as the unit of analysis, using the full counting method. To ensure meaningful visualization, a minimum document threshold of 20 per country was applied. A total of 31 countries met this threshold and were included in the network. Normalization was performed using the association strength method. In the visualization configuration, node weight represented document count, and clustering analysis was applied to identify distinct collaborative clusters. The bibliometrix R package was used to generate a world map depicting the geographical distribution and collaborative relationships of countries publishing on this topic. A minimum collaboration threshold (min edges = 2) was applied, meaning that only collaborative relationships with at least two co-authored documents were displayed as connecting lines. Bibliometrix was also used to identify the top 20 most productive countries based on corresponding author affiliations. A stacked bar chart was generated to display the proportion of single-country publications (SCP) versus multiple-country publications (MCP). SCP represents research conducted within a single country, while MCP indicates internationally collaborative projects led by that country. This analysis aimed to assess both research productivity and the extent of international collaboration for each country.

For institution-level analysis, VOSviewer was configured with co-authorship as the analysis type and organizations as the unit of analysis, using full counting. A minimum document threshold of 20 and a minimum citation threshold of 600 were applied to focus on high-productivity, high-impact institutions. A total of 56 organizations met these criteria and were included in the network. Normalization was performed using the association strength method. Node weight was set to represent document count, and clustering analysis was applied to identify collaborative clusters among these leading institutions.

#### 2.3.3 Journal analysis

To identify core journals and understand the intellectual structure of the research field, bibliographic coupling analysis and co-citation analysis were conducted. Bibliographic coupling occurs when two journals cite the same reference, suggesting shared research themes or academic backgrounds, and is useful for identifying currently active journals and their thematic clusters. Co-citation analysis reveals the foundational knowledge base and classic literature sources that have shaped the field. Together, these approaches provide a comprehensive view of both the current research landscape and its intellectual roots.

For the analysis of active journals, VOSviewer was configured to perform bibliographic coupling with sources as the unit of analysis. To focus on journals with substantial contributions to the field, a minimum document threshold of 10 publications per journal was established. A total of 68 journals met this criterion and were included in the network. Normalization was performed using the association strength method. In the resulting visualization, node size is proportional to the number of publications from each journal, and lines represent bibliographic coupling relationships. Colors indicate distinct clusters of journals sharing similar research themes or academic backgrounds. For the analysis of cited journals, VOSviewer was configured to perform co-citation analysis with cited sources as the unit of analysis. To ensure meaningful representation of foundational literature, a minimum citation threshold of 300 citations per cited journal was established. A total of 104 cited journals met this criterion and were included in the network. Normalization was performed using the association strength method. In the resulting visualization, node size is proportional to the total number of citations received from the analyzed publications, and lines represent co-citation links. Colors identify major disciplinary clusters that constitute the knowledge base of the field.

#### 2.3.4 Author analysis

To illustrate influential researchers and understand the collaborative and intellectual structures among authors in the field, three complementary analyses were conducted. Co-authorship analysis reveals collaborative relationships and identifies core research teams, while temporal overlay visualization adds a dynamic dimension by showing how author activity has evolved over the study period. Co-citation analysis uncovers the foundational contributors and intellectual connections that have shaped the field.

For the author co-authorship analysis, VOSviewer was configured with co-authorship as the analysis type and authors as the unit of analysis. To focus on researchers with substantial contributions and impact, a minimum document threshold of five publications and a minimum citation threshold of 200 citations per author were applied. From the initial dataset, 226 authors met these criteria, and the top 150 authors by publication count were selected for visualization to ensure clarity and interpretability of the network. Normalization was performed using the association strength method. In the resulting visualization, node size is proportional to the number of publications by each author, and lines represent co-authorship relationships. Colors indicate distinct clusters representing major research teams and their collaborative structures. To examine the temporal dynamics of author activity, an overlay visualization was generated using the same co-authorship network. Here, node colors represent the average publication year of each author’s documents: authors with earlier average publication years (2020 and before) appear in purple or dark blue, while those with more recent activity (2021 and after) appear in yellow or bright green. Node size continues to reflect publication volume, enabling simultaneous assessment of productivity and temporal activity patterns.

For the cited author co-citation analysis, CiteSpace was configured to analyze co-citation relationships among cited authors. The time span was set to 2016–2025 with one-year slices, and the selection criteria were set to the top 30 most cited authors per slice. After removing isolated nodes and unidentified entries, a total of 72 authors were included in the network. In the visualization, node size is proportional to the total citation count received from the analyzed publications. The tree-ring structure within each node represents the citation time distribution (2016–2025), with thicker rings indicating higher citation activity in corresponding years. Nodes with high betweenness centrality (>0.1) are highlighted with purple rings, identifying authors who serve as key intellectual bridges connecting different research clusters within the field.

#### 2.3.5 Core document and reference network analysis

At the document level, two complementary analyses were conducted to examine foundational publications and trace the evolution of research themes: bibliographic coupling of highly cited documents and co-citation analysis of cited references. Bibliographic coupling occurs when two documents cite the same reference, indicating thematic similarity in contemporary research and helping to identify currently influential papers and their clustering patterns. Co-citation analysis, where two documents are cited together by subsequent publications, reveals the intellectual foundation and classic works that have shaped the field. Citation burst detection was also applied to identify documents with sharp increases in citation activity, highlighting emerging research fronts and shifts in scholarly attention over time.

For the document-level bibliographic coupling analysis, VOSviewer was employed with bibliographic coupling as the analysis type and documents as the unit of analysis. A minimum citation threshold of 150 citations per document was established to focus on highly impactful publications, resulting in 63 documents meeting this criterion for network inclusion. Normalization was performed using the association strength method. In the resulting density visualization, each node corresponds to a specific document, with brightness and color temperature reflecting citation weight. Proximity between nodes indicates a high degree of shared references, revealing thematic similarities among contemporary studies.

The cited reference co-citation analysis was conducted using CiteSpace, with the time span set to 2016–2025 using one-year slices. The selection criteria were set to the top 10 most cited references per slice, yielding a network of 77 cited references. In the resulting visualization, node size reflects total citation frequency received from the analyzed publications. Each node contains a tree-ring structure representing the citation history from 2016 to 2025, where thicker rings indicate higher citation activity in corresponding years. Documents with high betweenness centrality (greater than 0.1) are highlighted with purple rings, identifying key intellectual bridges connecting different research clusters. Subsequently, citation burst detection was performed using CiteSpace to capture emerging research fronts. The 25 documents with the strongest citation bursts were selected for visualization, where red segments indicate burst duration and light blue lines represent the full 2016–2025 time span. Documents are ordered by burst start year to illustrate the temporal evolution of research focus and shifting patterns of scholarly attention throughout the study period.

#### 2.3.6 Research hotspots and thematic distribution analysis

To map the intellectual landscape and identify emerging trends in the field, a series of keyword-based analyses was conducted using CiteSpace. Keywords, as concise descriptors of research content, underwent data cleaning to ensure consistency: “deep brain stimulation(dbs)” was merged into “deep brain stimulation,” “disorder” was standardized to “disorders,” and “Parkinson disease” was corrected to “Parkinsons disease.” Additionally, irrelevant terms such as “botulinum toxin,” “classification,” and “diagnosis” were removed as they did not align with the study’s core focus. For keyword co-occurrence analysis, CiteSpace was configured with a 2016–2025 time span (one-year slices), keyword node type, and selection criteria of the top 50 most frequent keywords per slice. The network was pruned using the pathfinder algorithm and sliced network pruning to simplify its structure while preserving essential relationships, resulting in a final network of 135 keywords. In the visualization, node size reflects co-occurrence frequency, and the tree-ring structure within each node represents temporal distribution (2016–2025). Keywords with high betweenness centrality (> 0.1) are highlighted with purple rings, indicating bridging concepts across subfields.

Cluster analysis was performed to identify major research themes, with labels generated via the log-likelihood ratio (LLR) algorithm and clustering quality assessed using modularity Q and mean silhouette score. A timeline visualization tracked the evolution of these clusters, with horizontal lines representing distinct themes and keywords positioned by their first appearance year. Links between keywords illustrate co-occurrence relationships, revealing topic persistence or shifts over time. To capture shifting research frontiers, citation burst detection (minimum duration: 2 years) identified 38 keywords with significant bursts, with the top 25 visualized in chronological order. Red segments in the display indicate burst durations, highlighting periods of sudden scholarly attention and illustrating the sequential emergence of research hotspots from 2016 to 2025.

## 3 Results

### 3.1 Literature search and selection

**Figure 1:**
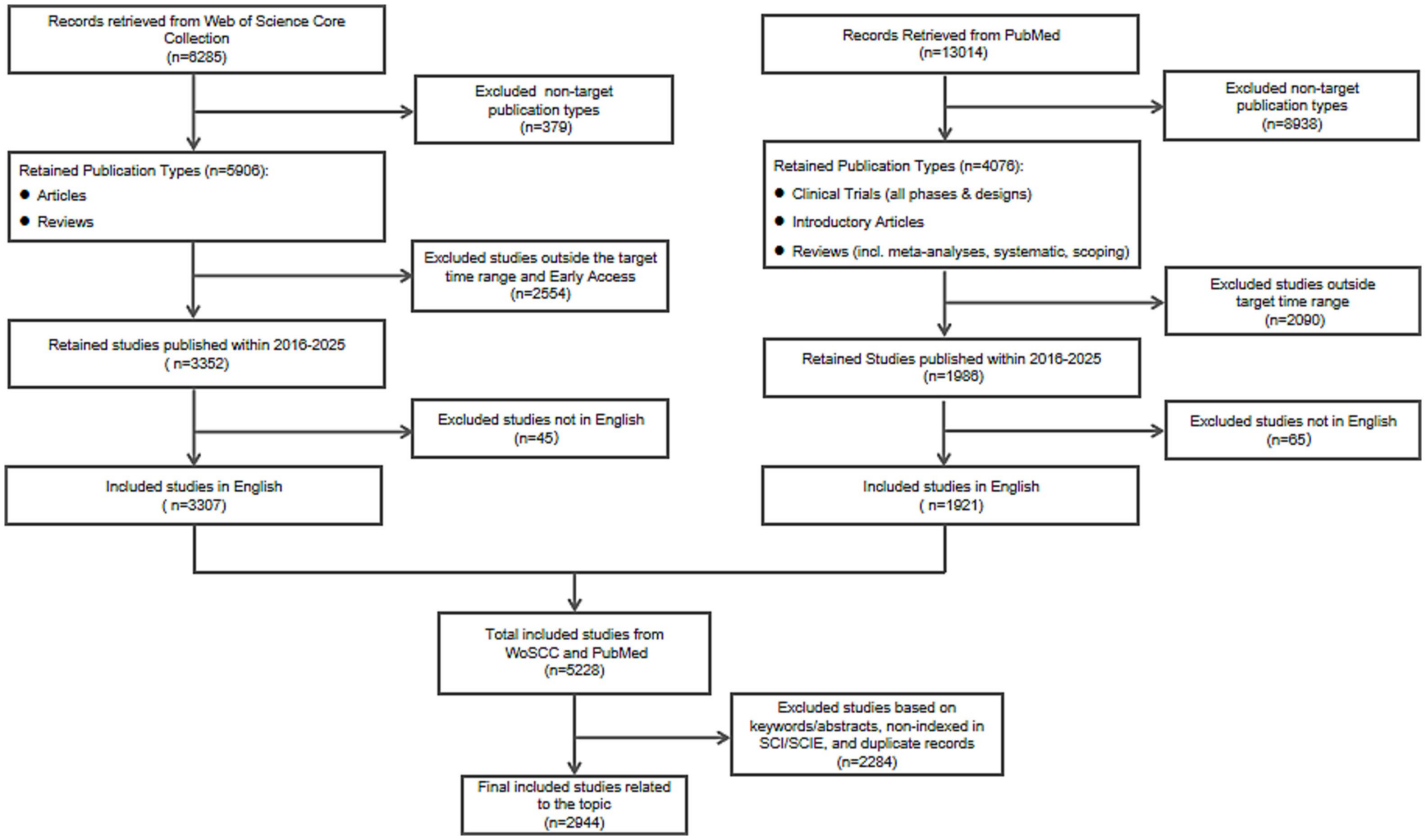
PRISMA flow diagram of the literature search and selection process from Web of Science Core Collection and PubMed.

A total of 6,285 records were retrieved from the WoSCC database. After screening, 3,307 studies were finally included. For PubMed, 13,014 records were initially retrieved, and 1,921 studies were ultimately included. Following the merging of records from both databases, duplicate entries were removed, and further screening was conducted, resulting in a final total of 2,944 studies that were ultimately included in the subsequent bibliometric analysis (Figure 1).

### 3.2 Leading countries/regions

**Figure 2:**
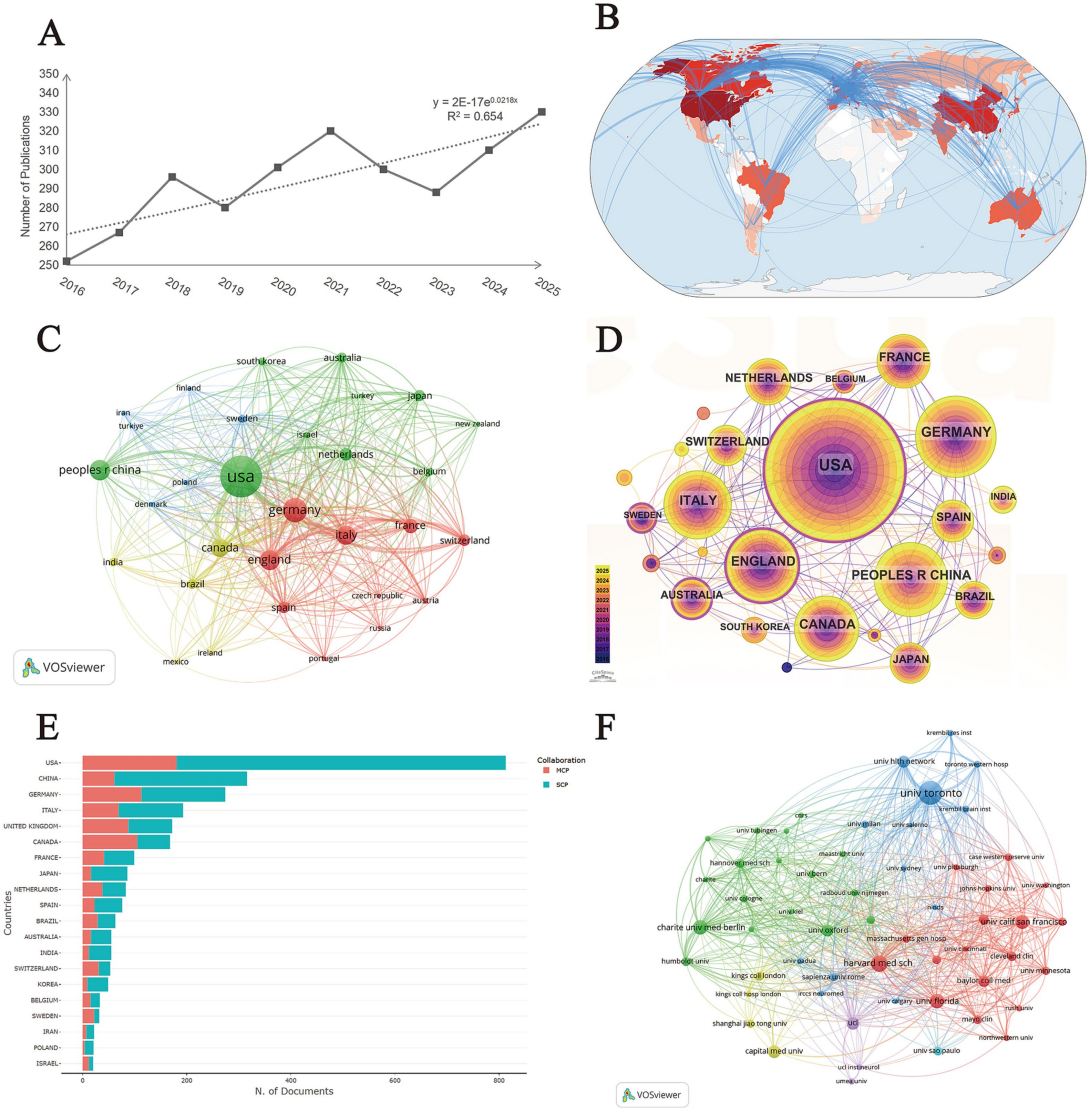
Number of publications, cooperation between countries and institutions. **(A)** Annual publication trends from 2016 to 2025. **(B)** Global geographical distribution and collaborative intensity of publications. The heatmap represents national productivity based on publication frequency, while the connecting lines illustrate the strength of international collaborative networks. The color density correlates with the volume of research output from each region. **(C)** Co-occurrence network map of high-productivity countries. Line thickness corresponds to the strength of collaborative ties between countries. **(D)** Citespace visualization of the leading countries in publication output for research on electrical stimulation for movement disorders. **(E)** Top 20 most productive countries of corresponding authors. The stacked bars illustrate the proportion of single country publications (SCP, blue) and multiple country publications (MCP, red). SCP represents internal research within a single country, while MCP indicates international collaborative efforts led by that country. **(F)** Collaborative network among institutions meeting the threshold of more than 20 publications and more than 600 citations.

Publication volume serves as a key indicator of the productivity and progress within a research field. As illustrated in Figure 2A, following the initial literature search in the Web of Science (WOS) core collection, a preliminary screening of the retrieved documents was conducted, and the number of publications per year was determined based on the “Final Publication Year” field.

The statistical results presented in Figure 2A indicate an overall upward trend in annual publication volume from 2016 to 2025, albeit accompanied by fluctuations. The volume increased steadily from 252 publications in 2016. Despite experiencing provisional temporary declines in 2019 and 2023, the field maintained a high level of activity. The publication count reached a stage peak in 2021 and ultimately culminated at its highest peak within the study period, at 330 articles, in 2025, collectively forming a sustained high output development phase.

To visualize the global collaborative landscape, a country cooperation map was generated using the ‘bibliometrix’ R package, applying a minimum threshold of two collaborations per country (Figure 2B). In this visualization, the depth of the red shading indicates the research output of each country, while the connecting lines represent specific inter-country partnerships. The results reveal a highly integrated network, with the most robust academic productivity and synergy concentrated in North America, Western Europe, and East Asia. This pattern underscores a globalized research framework where high-output nations act as central pillars, facilitating extensive cross-continental knowledge exchange.

A co-authorship network analysis of high-productivity nations was conducted using the full counting method, yielding a map of 31 nodes categorized into four distinct clusters (Figure 2C). The USA emerged as the dominant hub with the highest total link strength (849), followed by Germany (662) and England (579), indicating their central roles in global scientific collaboration. Notably, while China ranked third in terms of document volume (334), its total link strength (170) and citation count (4,885) were lower compared to several European nations with smaller output scales. This suggests a pattern where Western countries exhibit higher levels of international integrative research, whereas China’s productivity is currently characterized by a higher degree of intra-national focus and relatively sparse cross-border collaborative ties.

According to Figure 2D and Table 1, the United States (USA) dominates the research landscape with 1,099 publications and the highest betweenness centrality (BC) of 0.18. Betweenness centrality serves as a key metric for a node’s ‘brokerage’ capacity, with values exceeding 0.10 identifying pivot nodes that bridge different research clusters. Notably, while Sweden produced fewer publications (Kapadia et al., 2014), its high centrality (0.16) indicates its role as a critical international collaborator. Conversely, China exhibited a high volume of output (334) but a centrality of 0.00, suggesting that its research activities, despite their scale, remain relatively isolated or concentrated within localized clusters in the global cooperation network.

**Table 1:** Top ten countries by research output in electrical stimulation for movement disorders.

Rank	Country	Document	Centrality
1	USA	1,099	0.18
2	Germany	445	0.06
3	China	334	0.00
4	England	325	0.12
5	Italy	307	0.05
6	Canada	276	0.09
7	France	188	0.03
8	Netherlands	149	0.1
9	Spain	135	0.1
10	Switzerland	131	0.00

To further distinguish between local research capacity and international leadership, the distribution of corresponding authors’ countries was analyzed (Figure 2E). The USA significantly outpaced other nations with over 800 documents, maintaining a substantial volume of both SCP and MCP. Interestingly, while China ranked second in total output, it exhibited a relatively high proportion of SCP, suggesting a strong focus on domestic research initiatives. In contrast, European nations such as Germany, the United Kingdom, and Italy demonstrated higher ratios of MCP relative to their total output, underscoring their roles as central hubs for international multicenter collaborations. Furthermore, Canada showed a balanced profile with a high degree of international engagement, reflecting its pivotal position in the global neurosurgical and neuromodulation research network. This distinction between SCP and MCP highlights that while emerging regions are rapidly increasing their publication volume, established Western regions continue to act as primary facilitators of global cross-border scientific exchange in electrical stimulation for motor disorders.

### 3.3 Visualization of institution collaboration

Regarding institutional contributions (Table 2), the University of Toronto emerges as the global leader in this field, contributing 166 publications. Notably, it also demonstrates the most significant collaborative breadth, recording a Total Link Strength (TLS) of 278—a metric quantifying the aggregate intensity of cooperative ties between a specific institution and its peers. Harvard Medical School (96) and the University of Florida (83) follow as prominent contributors. Among European organizations, Charité University Medicine Berlin (79 papers, TLS 149) and UCL (66 papers, TLS 81) represent the core anchors in research on electrical stimulation for motor disorders.

**Table 2:** Top 10 institutions with the highest research output on electrical stimulation therapy for movement disorders.

Rank	Institution	Document	Total link strength
1	University of Toronto	166	278
2	Harvard Medical School	96	166
3	University of Florida	83	118
4	Charité—Universitätsmedizin Berlin	79	149
5	University of California, San Francisco	76	102
6	University College London	66	81
7	Capital Medical University	65	22
8	University Health Network‌	61	117
9	University of Oxford	61	93
10	Baylor College of Medicine	56	59

Rank: based on the publication count.

The institutional collaboration network (Figure 2F) further elucidates these structural interconnections. Our analysis underscores that the University of Toronto maintains the most extensive collaborative network, acting as the primary global nexus for electrical stimulation for motor disorders. Other key collaborative hubs include Harvard Medical School and Charité University Medicine Berlin, both of which exhibit high TLS, signifying their roles as critical intermediaries in global institutional knowledge exchange.

### 3.4 Visualization of journals and co-cited journals

**Figure 3:**
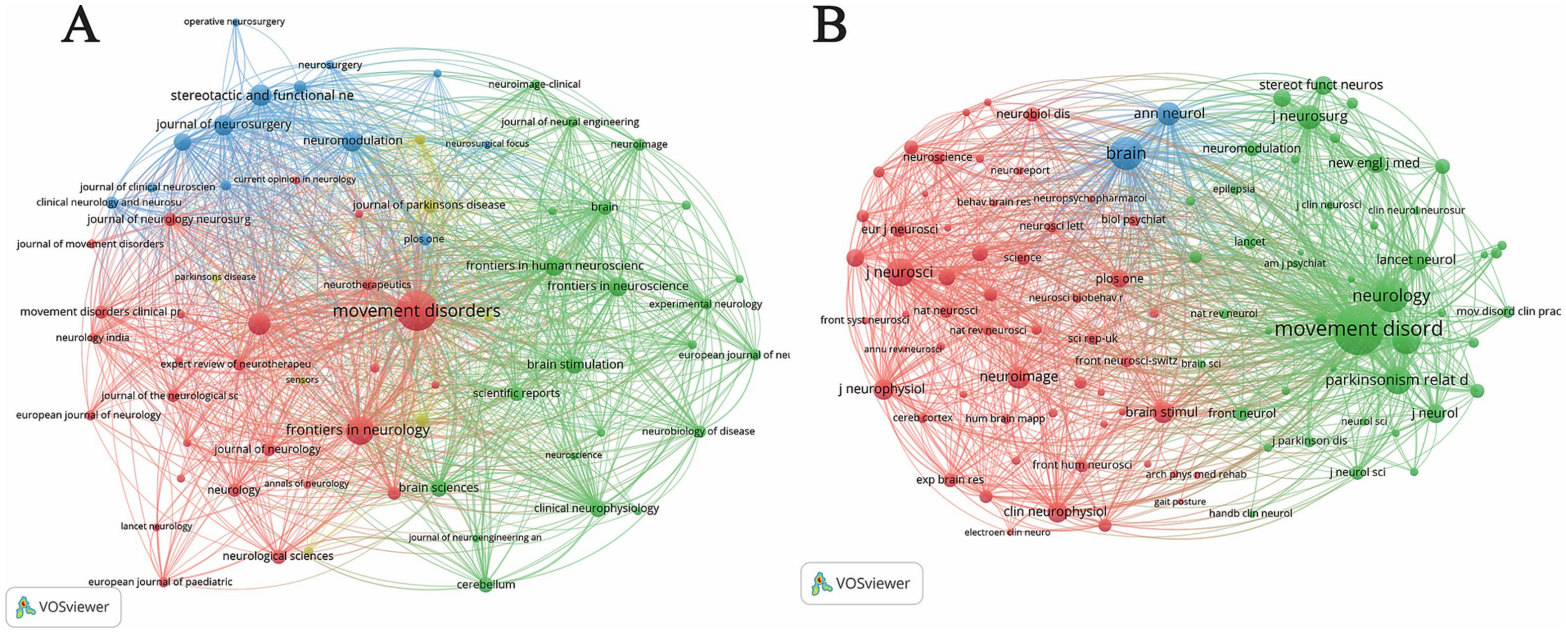
Journal analysis on electrical stimulation for movement disorders. **(A)** Bibliographic coupling network of active journals. Nodes represent journals, with node size proportional to the number of publications. Lines indicate bibliographic coupling ties (journals citing the same references). The colors represent distinct clusters of journals with similar research themes or academic backgrounds. A minimum threshold of 10 publications per journal was applied. **(B)** Co-citation network of cited sources. Nodes represent cited journals (minimum threshold: 300 citations), with size proportional to the total number of citations received within the dataset. Lines indicate co-citation links, where two journals are cited together in the same article. Colors denote major disciplinary clusters that constitute the knowledge base of the field.

To identify the core publication venues and their intellectual proximity, a bibliographic coupling analysis of journals was conducted (Figure 3A). Bibliographic coupling occurs when two journals cite the same third work, indicating shared research interests or theoretical frameworks. Among the 68 journals meeting the minimum threshold of 10 publications, *Movement Disorders* emerged as the most influential venue, leading in both publication volume (258 documents) and citation impact (10,132 citations), while maintaining the highest TLS (135,866) (Table 3).

**Table 3:** Top ten most productive journals in research on electrical stimulation for movement disorders.

Rank	Journal	Documents	Citations	TLS
1	Movement Disorders	258	10,132	135,866
2	frontiers in Neurology	129	2,088	57,759
3	Parkinsonism & Related Disorders	77	1,595	31,836
4	Stereotactic and Functional Neurosurgery	74	869	25,470
5	Journal of Neurosurgery	62	1,704	28,794
6	Neuromodulation	62	984	29,637
7	Frontiers in Neuroscience	59	1,678	28,131
8	Frontiers in Human Neuroscience	57	765	32,677
9	Brain Sciences	50	492	21,443
10	World Neurosurgery	50	466	24,980

Rank: based on the publication count. TLS, Total Link Strength.

The visualization map (Figure 3A) reveals several thematic clusters. The red cluster, centered around *Movement Disorders* and *Frontiers in Neurology*, primarily focuses on clinical phenomenology and neurological management. The blue cluster is dominated by neurosurgical and functional journals, such as *Stereotactic and Functional Neurosurgery* (74 documents) and *Journal of Neurosurgery* (62 documents), reflecting a focus on surgical interventions and electrode implantation techniques. Meanwhile, the green cluster, featuring *Brain Stimulation*, *Brain*, and *Frontiers in Human Neuroscience*, represents a multidisciplinary bridge between neurophysiology and advanced neuromodulation technologies. These results underscore that research on electrical stimulation for motor disorders is highly interdisciplinary, spanning clinical neurology, functional neurosurgery, and neuroengineering.

To explore the intellectual basis of the field, a journal co-citation analysis was conducted (Figure 3B). While bibliographic coupling identifies active contemporary venues, co-citation analysis highlights the foundational sources that underpin current research. Among the 104 journals meeting the threshold of 300 citations, *Movement Disorders* remained the most frequently cited source (15,448 citations, TLS 1,042,878), followed by *Neurology* (7,218 citations), *Brain* (6,967 citations), and *Parkinsonism & Related Disorders* (4,836 citations) (Table 4).

**Table 4:** Top ten co-cited journals in research on electrical stimulation for movement disorders.

Rank	Journal	Citations	TLS
1	Movement Disorders	15,448	1,042,878
2	Neurology	7,218	563,039
3	Brain	6,967	584,083
4	Parkinsonism and Related Disorders	4,836	360,742
5	Journal of Neurology, Neurosurgery and Psychiatry	4,690	361,896
6	Journal of Neuroscience	4,663	418,570
7	Annals of Neurology	3,209	283,426
8	Brain Stimulation	2,914	242,789
9	Journal of Neurophysiology	2,687	236,922
10	Clinical Neurophysiology	2,597	252,973

Rank: based on the citation count. TLS: Total Link Strength.

The co-citation network (Figure 3B) is organized into three primary clusters. The green cluster represents the clinical core, dominated by high-impact neurology and movement disorder journals, which provide the essential clinical evidence and diagnostic guidelines. The blue cluster, featuring *Annals of Neurology* (3,209 citations) and *Brain*, signifies the foundational neurobiological and pathological research. The red cluster, centered around *Journal of Neuroscience* (4,663 citations), *NeuroImage*, and *Clinical Neurophysiology*, reflects the integration of neurophysiological mechanisms and neuroimaging evidence. The dense inter-cluster connections, particularly between *Movement Disorders* and *Brain*, illustrate a robust synergy between clinical practice and fundamental neuroscience in the evolution of electrical stimulation therapies.

### 3.5 Visualization of authors and co-cited authors

**Figure 4:**
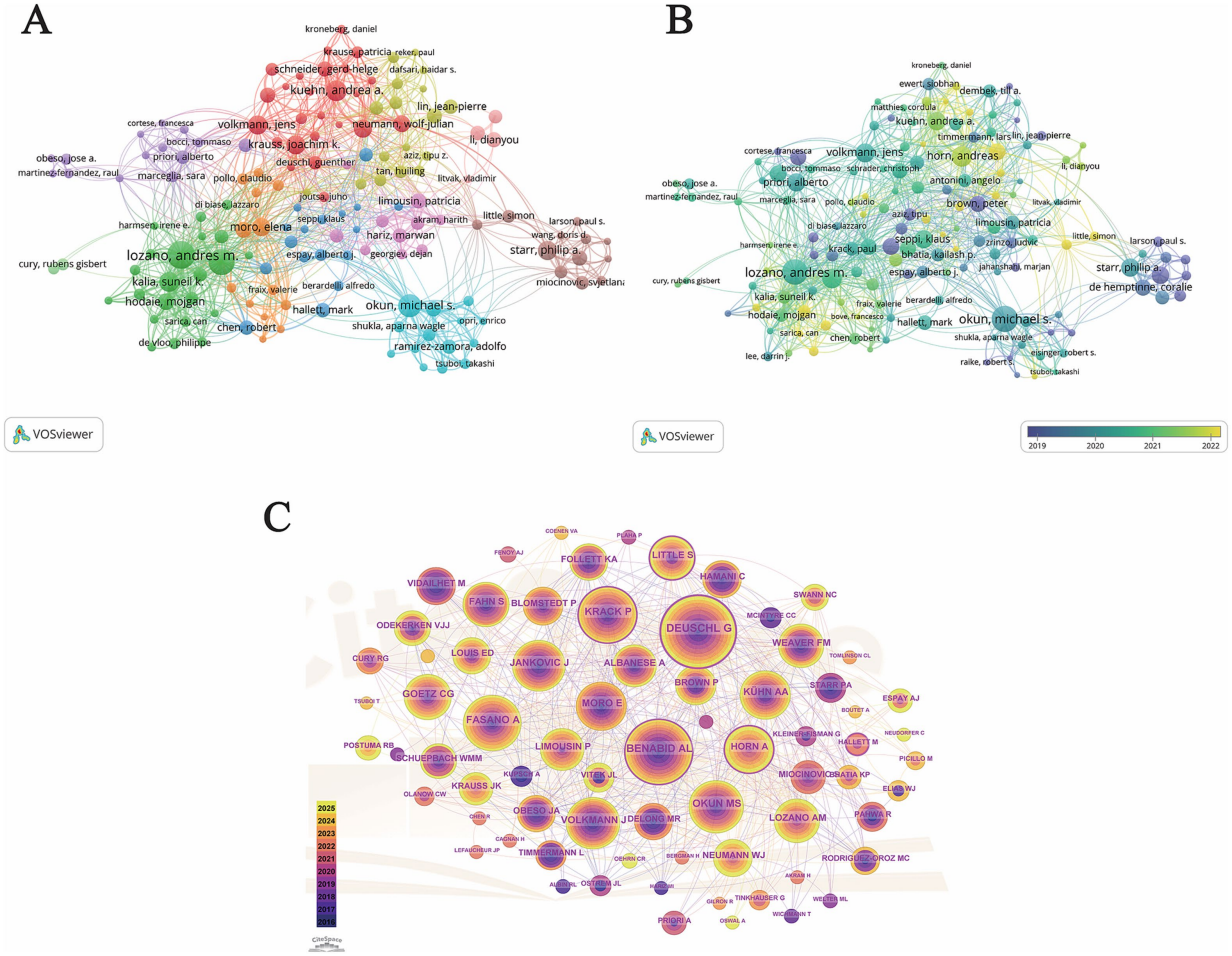
Analysis of influential authors in research on electrical stimulation for movement disorders. **(A)** Author collaboration network. Nodes represent authors (minimum threshold: 5 publications and 200 citations), with size proportional to the number of documents. Lines indicate co-authorship ties. The top 150 authors are visualized and divided into 11 thematic clusters (denoted by different colors), representing major research teams and their collaborative structures. **(B)** Overlay visualization of the author collaboration network. Nodes represent authors, with colors indicating their average publication year. Purple/dark blue nodes represent authors active in the early stages of the sampled period (circa 2020 and before), while yellow/bright green nodes highlight authors with more recent research activity (circa 2021 and beyond). The node size reflects the publication volume **(C)** Co-citation network of cited authors. Nodes represent cited authors (Top 30 per time slice), with node size proportional to the total number of citations received. The tree-ring structure within each node reflects the temporal distribution of citations from 2016 to 2025. Purple rings highlight nodes with high betweenness centrality (> 0.1), indicating their role as key intellectual bridges.

To identify the leading researchers and their collaborative ecosystems, an author co-authorship network was constructed (Figure 4A). After applying a threshold of at least 5 publications and 200 citations, the top 150 authors were visualized, revealing 11 distinct clusters that represent the core research teams in the field. As shown in Table 5, Lozano, Andres M. (71 documents, 3,843 citations, TLS 294) and Fasano, Alfonso (64 documents, 2,262 citations, TLS 206) emerged as the most prolific and influential scholars, both demonstrating exceptionally high TLS values. They are followed by other prominent experts such as Okun, Michael S. (50 documents, 3,990 citations) and Kuehn, Andrea A. (40 documents, 1,996 citations).

**Table 5:** Top ten most productive authors in research on electrical stimulation for movement disorders.

Rank	Author	Document	Citations	TLS
1	Lozano, Andres M.	71	3,843	294
2	Fasano, Alfonso	64	2,262	206
3	Okun, Michael S.	50	3,990	160
4	Kuehn, Andrea A.	40	1,996	119
5	Kalia, Suneil K.	38	1,093	177
6	Krauss, Joachim K.	35	1,340	103
7	Starr, Philip A.	34	2,017	103
8	Volkmann, Jens	34	2,137	142
9	Horn, Andreas	32	2,488	108
10	Moro, Elena	31	1,600	151

Rank: based on the document count. TLS, Total Link Strength.

The collaborative landscape is characterized by high-density clusters centered around these world-renowned authorities. The green cluster is spearheaded by Lozano and Fasano, reflecting a dominant North American research group (primarily based in Toronto) with the most extensive international collaborative reach. The cyan cluster, led by Okun, represents a major clinical research hub in the United States. Furthermore, the red cluster, featuring Kuehn (40 documents) and Volkmann (34 documents), underscores a robust European collaborative axis. The presence of 11 clusters highlights a diverse yet highly interconnected intellectual community, where leading scholars act as central nodes facilitating large-scale multi-center studies and knowledge dissemination in electrical stimulation for motor disorders.

The temporal evolution of author contributions was further examined using an overlay visualization (Figure 4B), where the node color represents the average publication year. The results reveal a clear transition in the field’s leadership and research focus over time. Highly influential scholars such as Lozano, Starr, Philip A., and Brown, Peter appear in teal and blue-purple hues, indicating their foundational and sustained contributions since the early phase of the study period (average year around 2019–2020). Conversely, a cluster of ‘emerging leaders’ is identified by yellow and bright green nodes, signifying high research activity in more recent years (2022 and beyond). Notably, authors such as Horn, Kuehn, and Hodaie occupy the yellow-coded regions. Their recent prominence is largely associated with cutting-edge advancements in neuroimaging-based electrode localization and adaptive stimulation technologies. This temporal shift underscores a move from traditional clinical outcome studies toward more technologically integrated and personalized neuromodulation strategies, led by a new generation of researchers collaborating closely with established authorities.

To explore the foundational intellectual structure of the field, an author co-citation analysis was conducted using CiteSpace (Figure 4C). Betweenness centrality and citation counts were utilized to identify the most influential scholars whose works constitute the knowledge base of electrical stimulation for motor disorders. As presented in Table 6, Deuschl G (544 citations, centrality 0.32) and Benabid AL (426 citations, centrality 0.16) emerged as the most prominent authorities, both exhibiting high citation counts and significant centrality. Notably, Benabid AL is widely recognized as the pioneer of DBS, and his central position reflects the enduring impact of his foundational discoveries.

**Table 6:** Top ten co-cited authors with the most citations in research on electrical stimulation for movement disorders.

Rank	Author	Citations	Centrality
1	Deuschl G	544	0.32
2	Benabid AL	426	0.16
3	Krack P	342	0.13
4	Fasano A	340	0.08
5	Okun MS	327	0.04
6	Volkmann J	304	0.07
7	Jankovic J	292	0.01
8	Kuhn AA	269	0.06
9	Moro E	263	0.05
10	Goetz CG	259	0.07

Rank: based on the citation count.

The network (Figure 4C) reveals several ‘pivot nodes’ characterized by prominent purple rings, which signify their crucial role in connecting different research themes. For instance, Krack P (342 citations, centrality 0.13) and Little S (213 citations, centrality 0.12) act as major intellectual bridges. Furthermore, the emergence of more recent influential figures, such as Horn A (244 citations, centrality 0.11) and Neumann WJ (162 citations, centrality 0.06), reflects the field’s evolving focus toward network-based neuroimaging and adaptive neuromodulation. The high density of connections (491 links) among these 72 cited authors underscores a well-established and highly integrated theoretical framework supporting current advancements in motor disorder therapies.

### 3.6 Bibliographic coupling analysis

**Figure 5:**
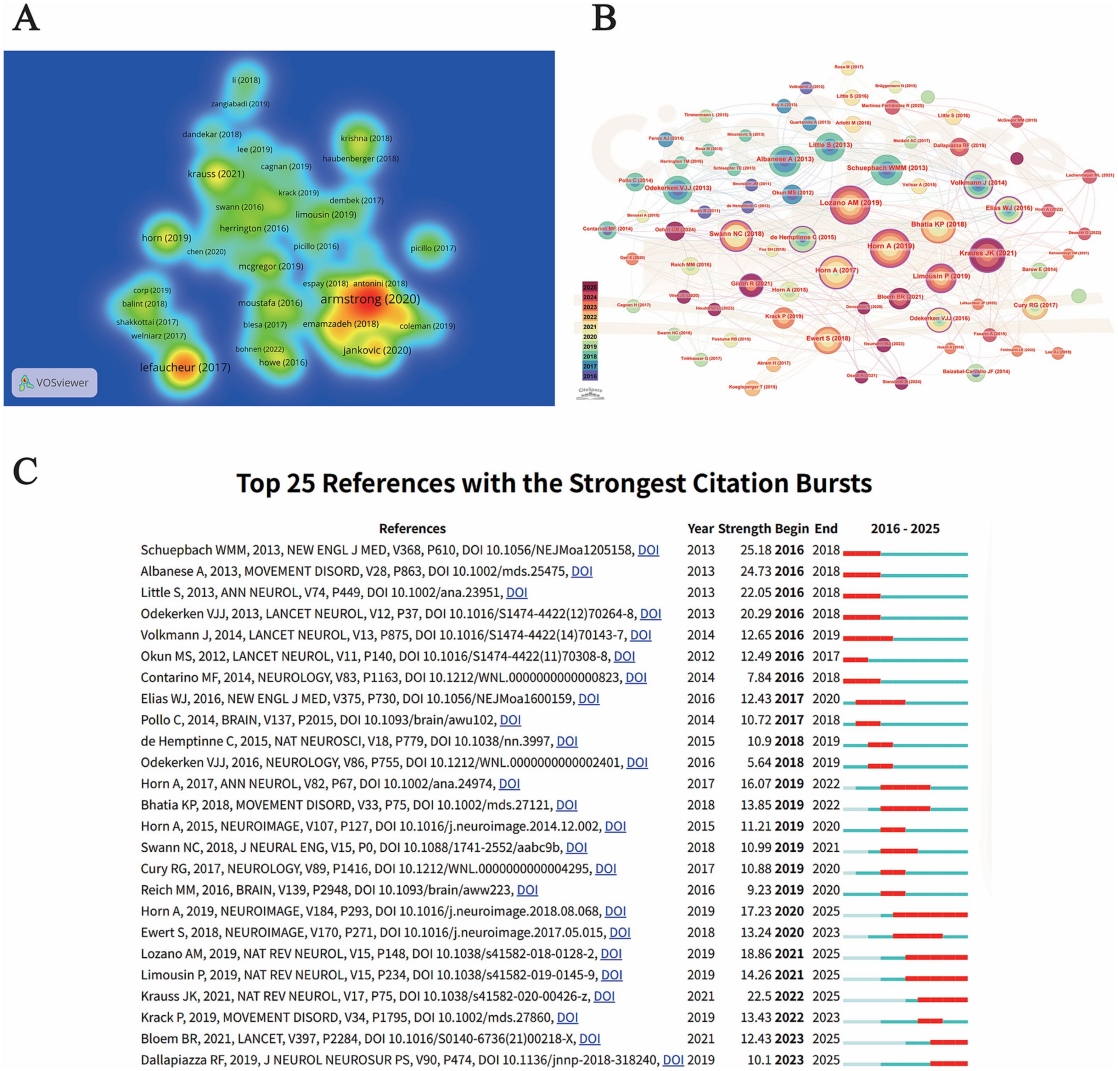
Bibliographic analysis of core documents and reference networks in motor disorder electrical stimulation research. **(A)** Density visualization of the document network. Each node represents a specific document (minimum threshold: 150 citations), with brightness and color heat indicating the citation weight. The network consists of 63 documents divided into 9 thematic clusters. Proximity between nodes suggests a high degree of shared references, reflecting thematic similarity in contemporary research. **(B)** Co-citation network of cited references. Nodes represent cited references (Top 10 per time slice), with node size proportional to the total co-citation frequency. The tree-ring structure within nodes reflects the citation history from 2016 to 2025. Purple rings highlight references with high betweenness centrality (> 0.1). **(C)** Top 25 references with the strongest citation bursts. The red bars indicate the duration of the citation burst, while the light blue lines represent the entire period from 2016 to 2025. References are ranked by the onset year of the burst to illustrate the temporal transition of research focuses.

A bibliographic coupling analysis was performed to elucidate the intellectual overlaps and contemporary research hotspots (Figure 5A). Among the 63 documents meeting the citation threshold, Armstrong and Okun (2020) and Lefaucheur et al. (2016) stood out as the most prominent nodes in terms of citation impact, with 2,094 and 1,301 citations, respectively (Table 7). Their positions as high-intensity heat centers in the density map underscore their roles as foundational clinical guidelines within the field. Interestingly, while the aforementioned papers lead in raw citations, documents such as Herrington et al. (2016) (TLS 378) and Krack et al. (2019) (TLS 322) exhibited the highest TLS. This disparity suggests that although guideline-based papers receive widespread individual citations, the works by Herrington and Krack serve as the primary ‘intellectual bridges’ that share extensive references with other contemporary studies.

**Table 7:** Top 10 documents with the highest citation impact in research on electrical stimulation for motor disorders.

Rank	Document	Citations	TLS
1	Armstrong (2020)	2094	46
2	Lefaucheur (2017)	1,301	39
3	Jankovic (2020)	807	8
4	Seppi (2019)	725	68
5	Krauss (2021)	607	135
6	Fox (2018)	600	92
7	Horn (2019)	592	57
8	Mcgregor (2019)	445	129
9	Howe (2016)	428	7
10	Emamzadeh (2018)	427	45

Rank: based on the citation count. TLS, Total Link Strength.

A reference co-citation analysis was performed using CiteSpace to map the intellectual foundation of this research domain (Figure 5B). Reference co-citation analysis identifies seminal works that are frequently cited together by subsequent studies, representing the foundational knowledge base. As summarized in Table 8, the network is anchored by several landmark publications. Lozano et al. (2019) and Horn et al. (2019) emerged as the most frequently cited references, with 87 and 86 co-citations respectively, highlighting their roles as modern pillars of electrical stimulation research. Notably, Swann Nicole et al. (2018) exhibited the highest betweenness centrality (0.43), serving as a critical ‘knowledge bridge’ that integrates diverse thematic clusters (Table 8).

**Table 8:** Top 10 most frequently co-cited references in the knowledge base of electrical stimulation for motor disorders.

Rank	Cited reference	Citations	Centrality
1	Lozano AM, 2019	87	0.19
2	Horn A, 2019	86	0.12
3	Bhatia KP, 2018	73	0.04
4	Krauss JK, 2021	67	0.21
5	Horn A, 2017	60	0.21
6	Schuepbach WMM, 2013	59	0.03
7	Albanese A, 2013	58	0
8	Swann NC, 2018	53	0.43
9	Little S, 2013	52	0.02
10	Limousin P, 2019	50	0.26

Rank: based on the citation count.

The temporal distribution of citations, visualized through the node tree-rings, indicates a shift in focus. Earlier landmark papers like Schuepbach et al. (2013) and Simon et al. (2013) established the clinical and adaptive paradigms of DBS. In contrast, more recent high-impact works, such as Krauss et al. (2021) and Horn et al. (2017) both with a centrality of 0.21, represent the evolving frontier toward individualized connectomics and normative brain atlases.

To identify the most influential papers during specific periods, a citation burst analysis was conducted on the co-cited references (Figure 5C). The results reveal a clear chronological transition in research themes. During the early stage (2016–2018), clinical trials and diagnostic guidelines exhibited the strongest bursts, led by Schuepbach et al. (2013) and Alberto et al. (2013), which established the early clinical framework for DBS.

In the mid-term phase (2019–2022), the focus shifted toward neuroimaging and mechanism-oriented studies, as evidenced by the high burst strength of Horn et al. (2017) and Bhatia et al. (2018). Notably, the most recent and ongoing bursts (ending in 2025) are dominated by papers such as Lozano et al. (2019), Krauss et al. (2021), and Bloem et al. (2021). The persistent bursts of these recent works, particularly those focused on connectomics Horn et al. (2017) and contemporary therapeutic reviews Krauss et al. (2021), suggest that neuroimaging-integrated neuromodulation and evidence-based personalized therapy will continue to be the primary research directions in the coming years.

### 3.7 Visualization of keywords co-occurrence, evolution and burst

**Figure 6:**
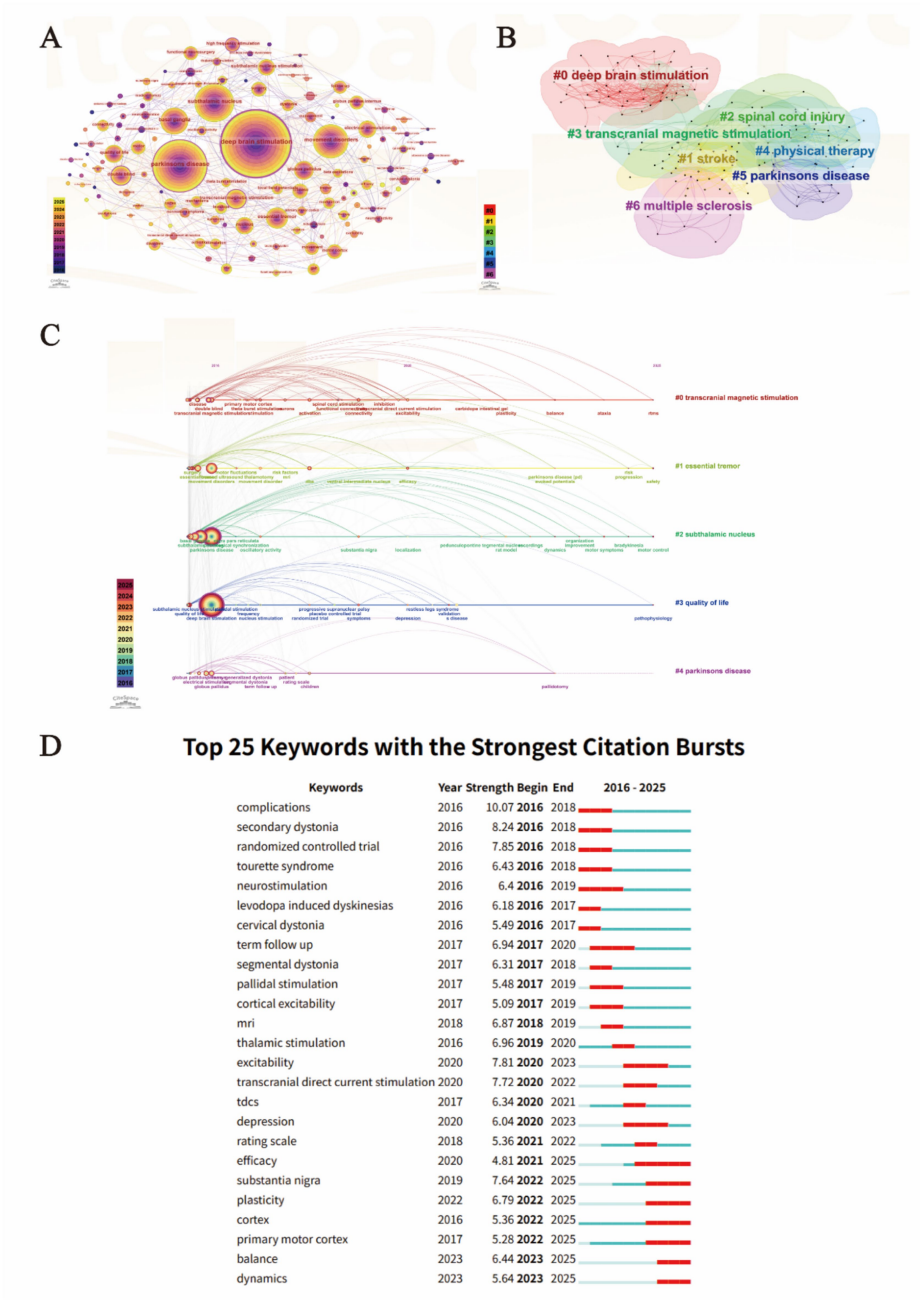
Analysis of research hotspots and thematic distribution in electrical stimulation for motor disorders. **(A)** Keyword co-occurrence network. Nodes represent keywords (Top 50 per time slice), with size proportional to the co-occurrence frequency. The tree-ring structure reflects the temporal distribution of keywords from 2016 to 2025. Purple rings highlight keywords with high betweenness centrality (> 0.1), indicating pivotal research themes that connect different sub-domains. **(B)** Cluster visualization of keywords. Different colored regions represent distinct thematic clusters, with Modularity Q = 0.3101 and Mean Silhouette S = 0.7031, indicating a significant and convincing cluster structure. **(C)** Timeline view of keyword clusters. Each horizontal line represents a distinct research cluster, with keywords plotted according to their initial appearance year. The lines between keywords represent their co-occurrence relationships. This visualization highlights the longitudinal evolution and longevity of specific research themes from 2016 to 2025. **(D)** Top 25 keywords with the strongest citation bursts. The red segments represent the duration of the burst, indicating the period when the keyword received sudden and intense academic attention. Keywords are sorted by their starting year to illustrate the chronological evolution of research frontiers from 2016 to 2025.

To identify the core research hotspots and thematic evolution, a keyword co-occurrence analysis was conducted using CiteSpace (Figure 6A). The network, comprising 135 nodes and 446 links, exhibits a modularity Q of 0.3101 and a weighted mean silhouette S of 0.7031, suggesting a reliable and significant cluster structure. As shown in Table 9, ‘deep brain stimulation’ (Count: 1,873, Centrality: 0.34) and ‘Parkinsons disease’ (Count: 1,175, Centrality: 0.11) emerged as the most prominent keywords, serving as the primary research anchors in this domain.

**Table 9:** Top ten most frequent keywords in research on electrical stimulation for movement disorders.

Rank	Keyword	Count	Centrality
1	Deep brain stimulation	1873	0.34
2	Parkinsons disease	1,175	0.11
3	Subthalamic nucleus	664	0.1
4	Movement disorders	570	0.04
5	Basal ganglia	369	0.07
6	Essential tremor	260	0.09
7	Transcranial magnetic stimulation	222	0.13
8	Globus pallidus	216	0.09
9	Double blind	203	0.19
10	Quality of life	187	0.06

Rank: based on the keyword’s occurrence count.

The analysis of betweenness centrality identifies several pivotal nodes that act as conceptual bridges within the field. As shown in Table 9, keywords such as ‘double blind’ (Centrality: 0.19), ‘transcranial magnetic stimulation’ (Centrality: 0.13), and ‘high frequency stimulation’ (Centrality: 0.12) demonstrate high centrality, underscoring the importance of rigorous clinical trial design and diverse neuromodulation modalities. Furthermore, keywords such as ‘subthalamic nucleus’ (Count: 654, Centrality: 0.1) and ‘basal ganglia’ represent the anatomical focus of electrical interventions. The dense interconnectedness of these keywords reflects a mature research landscape where clinical applications for movement disorders are deeply integrated with neurophysiological investigations and controlled clinical methodologies.

To further clarify the internal structure of these research hotspots, a keyword clustering analysis was performed (Figure 6B). The network yielded a Modularity Q of 0.3101 and a weighted mean Silhouette S of 0.7031, confirming that the partition of the research domain is both statistically significant and highly consistent. The keywords are primarily organized into five major clusters: #0 transcranial magnetic stimulation, #1 essential tremor, #2 subthalamic nucleus, #3 quality of life, and #4 Parkinsons disease, which collectively define the spatial landscape of current research.

The temporal evolution of research themes was further elucidated through a keyword timeline visualization (Figure 6C). While Cluster #2 (subthalamic nucleus) and Cluster #4 (Parkinsons disease) represent long-standing foundational pillars with continuous activity, the timeline reveals a clear shift in research focus within other clusters. For instance, Cluster #0 (transcranial magnetic stimulation) shows a significant expansion toward more diverse topics in recent years, such as ‘plasticity’, ‘carbidopa intestinal gel’, and ‘rtms’, indicating a broadening of the technological scope. Similarly, Cluster #1 (essential tremor) highlights a recent shift toward ‘risk progression’ and ‘safety’ profiles. Cluster #3 (quality of life) reflects a growing integration of patient-centered outcomes with clinical parameters. The sustained density of nodes and links across these timelines underscores the continuous innovation and the interdisciplinary nature of electrical stimulation research, moving from anatomical targeting toward functional and holistic patient management.

To detect the dynamic shifts in research focus, a keyword burst analysis was performed (Figure 6D). The results illustrate three distinct evolutionary phases of electrical stimulation for motor disorders. In the early phase (2016–2018), bursts were dominated by clinical safety and specific indications, such as ‘complications’ (Strength: 10.07), ‘secondary dystonia’ (8.24), and ‘randomized controlled trial’ (7.85), reflecting an era focused on establishing therapeutic efficacy and safety standards.

The mid-term phase (2019–2022) saw a surge in interest regarding non-invasive modalities and non-motor symptoms, with keywords like ‘transcranial direct current stimulation’ (7.72) and ‘depression’ (6.04) exhibiting significant bursts. Notably, the most recent phase (2022–2025) highlights an ongoing transition toward neurobiological mechanisms and functional integration. Keywords such as ‘substantia nigra’ (7.64), ‘plasticity’ (6.79), ‘cortex’ (5.36), and ‘primary motor cortex’ (5.28) have all shown persistent bursts continuing into 2025. Specifically, the emerging focus on ‘balance’ (6.44) and ‘dynamics’ (5.64) since 2023 represents the current research frontier, indicating a paradigm shift toward deciphering complex neural circuit dynamics and addressing refractory axial symptoms in neuromodulation therapies.

## 4 Discussion

This study employed CiteSpace, VOSviewer, and the bibliometrix R package to perform a visual analysis of literature related to electrical stimulation for movement disorders, retrieved from the Web of Science Core Collection and PubMed databases spanning the period from 2016 to 2025. While traditional systematic reviews typically focus on specific clinical outcomes or single interventions, our bibliometric approach constructs a macroscopic evolutionary trajectory of the entire field. Overall, the findings reveal a major paradigm shift in therapeutic strategies. In the early phase of our study timeframe, research primarily focused on validating the clinical efficacy and safety of foundational invasive techniques, particularly deep-brain stimulation for Parkinson’s disease. In contrast, recent trends indicate a strategic transition toward integrating neuroimaging and connectomics to enable personalized interventions. Correspondingly, there is a growing emphasis on elucidating specific neural network dynamics and plasticity to better understand and manage refractory axial symptoms such as balance disorders.

### 4.1 Bibliometric analysis of electrical stimulation for motor disorders: publications, collaboration, and influence trends

Analysis of publication volume indicates a robust and maturing research landscape. The United States currently dominates the field, ranking first in both publication volume and global network influence. Interestingly, while China ranks third in total publications, its exceedingly low centrality score exposes a significant structural imbalance within the global research network. This striking contrast suggests that although domestic funding and clinical interest in neuromodulation are surging in emerging regions, their research ecosystems remain largely isolated and highly dependent on single-country publications. The lack of international connectivity constitutes a major bottleneck, thereby hindering the widespread clinical generalization of novel protocols. To overcome this limitation and establish globally standardized intervention strategies, future research must prioritize breaking down geographical barriers and fostering international consortia, similar to the highly successful multicenter collaborative networks observed in European nations.

Furthermore, institutional and journal analyses reveal a highly mature and interconnected disciplinary ecosystem. The University of Toronto emerged as the undisputed global nexus, exhibiting the highest publication volume and the most extensive collaborative breadth. In addition, institutions such as Harvard Medical School and Charité University Medicine Berlin act as critical hubs driving high-impact international research. This consolidation of high-quality research is mirrored in the journal distribution. The journal Movement Disorders leads in both volume and co citation frequency, acting as the primary intellectual bridge connecting clinical neurology with functional neurosurgery and neuroengineering. The prominence of such top-tier multidisciplinary journals indicates that electrical stimulation for movement disorders has successfully transitioned into a highly integrated mainstream clinical science.

Visualization analysis of author collaboration patterns reveals dynamic research teams driven by core investigators. The field is anchored by prolific scholars such as Lozano AM and Fasano A, who maintain the most extensive international collaborative networks. A crucial temporal shift is also observed in the leadership and research focus. While the foundation of the field relies on the enduring impact of clinical pioneers, a new vanguard of researchers, including Horn A and Kuehn AA, has recently emerged at the forefront. Their recent prominence aligns perfectly with current research hotspots, specifically cutting-edge advancements in neuroimaging-based electrode localization and adaptive stimulation technologies. This trajectory highlights a broader transition from utilizing stimulation solely for symptom suppression toward applying precision neuromodulation guided by personalized brain atlases (Horn et al., 2019).

### 4.2 Analysis of research hotspots in electrical stimulation for motor disorders

#### 4.2.1 Temporal evolution of research hotspots transitioning from technical validation to mechanistic exploration

The temporal evolution of keywords and citation bursts delineates a distinct knowledge shift tracing a pathway from clinical validation to mechanistic exploration and individualized neuromodulation. In the early phase from 2016 to 2018, research was predominantly clinically driven. Prominent citation bursts for terms such as complications, secondary dystonia, and randomized controlled trial emphasized the primary necessity of establishing surgical safety and baseline therapeutic parameters for invasive interventions (Schuepbach et al., 2013; Alberto et al., 2013). Subsequently, between 2019 and 2022, the focus shifted significantly toward noninvasive modalities and the management of nonmotor symptoms. This transition is clearly evidenced by the strong bursts of interest in transcranial direct current stimulation and depression (Lefaucheur et al., 2016). The most recent evolutionary phase, extending through 2025, highlights an ongoing paradigm shift toward neurobiological mechanisms and functional network integration. The persistent emergence and sustained bursts of keywords, including plasticity, substantia nigra, balance, and dynamics, indicate that contemporary researchers are actively decoding complex neural circuit dynamics (Herrington et al., 2016; Lozano et al., 2019). This reflects a critical move toward developing highly personalized therapeutic protocols aimed at addressing refractory axial symptoms and driving genuine functional recovery.

#### 4.2.2 Current knowledge architecture: integrating deep brain stimulation with noninvasive neuromodulation

Keyword co-occurrence and clustering analyses reveal a well-defined and robust knowledge structure characterized by highly interconnected research themes. The analytical results confirm the reliability of the thematic clustering, indicating that the field has matured into five coherent subdomains. The contemporary research landscape is firmly anchored by the central pillars of DBS for Parkinson’s disease and the subthalamic nucleus (Armstrong and Okun, 2020). However, the robust expansion into independent clusters focusing on transcranial magnetic stimulation, essential tremor, and quality of life illustrates a dynamic disciplinary diversification. This specific knowledge architecture directly mirrors current clinical demands. As invasive anatomical targeting techniques have become standardized, researchers are strategically expanding their scope to incorporate holistic patient-centered outcomes and noninvasive modalities. This expansion has successfully created a comprehensive multidisciplinary ecosystem that seamlessly bridges functional neurosurgery with advanced neurorehabilitation science (Krack et al., 2019).

#### 4.2.3 Intellectual cornerstones and the lasting impact of foundational publications on current research

Within this established knowledge architecture, highly cited foundational literature provides the essential conceptual linkages that connect the diverse research clusters. Comprehensive guidelines and major clinical trials authored by leading scholars serve as structural anchors with massive citation impacts, establishing standard clinical protocols (Alberto et al., 2013; Bhatia et al., 2018). Concurrently, highly centralized publications act as critical intellectual bridges that integrate distinct subdomains. These foundational texts are universally utilized by researchers developing invasive surgical interventions as well as those exploring noninvasive rehabilitation therapies (Jakobs et al., 2019). By providing concrete clinical standards and elucidating core neurobiological mechanisms, these seminal works successfully guided the broader research community away from early isolated exploratory approaches (Lozano et al., 2019). Consequently, modern scientists consistently rely on these standardized frameworks to precisely measure how electrical stimulation rewires neural circuits and drives lasting motor recovery.

### 4.3 Summarized clinical insights and intervention strategies

While bibliometric analyses inherently focus on macroscopic research trends, summarizing the prominent intervention strategies identified in the core literature provides critical practical implications for clinicians. The overall evolutionary trajectory reveals two distinct but highly complementary clinical treatment strategies. First, in the realm of invasive neuromodulation, the historically established protocol of continuous open-loop stimulation is undergoing significant technological refinement. Driven by recent neurophysiological insights and the urgent need to address complex axial symptoms, current high-impact literature emphasizes a strategic transition toward directional stimulation and adaptive closed-loop systems (Krauss et al., 2021; Walid et al., 2019). These advanced interventions rely on real-time neurophysiological biomarkers such as local field potentials and cortical narrowband gamma oscillations to dynamically adjust stimulation parameters, thereby optimizing symptom relief while actively mitigating adverse network effects (Swann Nicole et al., 2018; Simon et al., 2013). Second, regarding noninvasive modalities, the literature robustly supports the integration of techniques such as transcranial direct current stimulation to manipulate cortical excitability and innate neuroplasticity actively (Lefaucheur et al., 2016). The most effective contemporary clinical strategies avoid deploying electrical stimulation in isolation. Instead, they systematically combine advanced neuromodulation with targeted physical therapies to maximize functional recovery and improve overall quality of life in patients with movement disorders (Elkouzi et al., 2019).

### 4.4 Limitations of the study

This study presents several inherent limitations commonly associated with bibliometric methodologies that must be clearly acknowledged.

First, regarding data acquisition, the bibliometric analysis was based on publications retrieved from the Web of Science Core Collection and PubMed, two of the most authoritative and widely used databases in the biomedical field. While this combination ensures comprehensive coverage of the core literature, the inclusion of additional databases such as Scopus or Embase might further expand the scope of retrieved publications. In addition, restricting the search strategy solely to English literature inevitably introduces linguistic and geographic selection biases, thereby potentially overlooking significant regional clinical advancements published in other languages.

Besides, the bibliometric methodology itself carries specific analytical constraints. The field is intrinsically susceptible to temporal citation lag, where recently published breakthrough studies have not yet accumulated sufficient citation frequencies to accurately reflect their true scientific significance within the visualization networks. Additionally, the visualization software tools operate strictly on objective mathematical counting and concurrent occurrence metrics. While this macroscopic quantitative approach ensures statistical rigor, it entirely lacks the capacity to evaluate the specific scientific context or the underlying sentiment of individual citations. Consequently, these network metrics cannot replace the nuanced qualitative appraisal of complete manuscripts.

Beyond the methodological constraints discussed above, the temporal scope of the analysis introduces additional limitations. The bibliometric analysis was restricted strictly to the recent ten-year period from 2016 to 2025. We explicitly acknowledge that focusing on this specific ten-year window is a heuristic analytical decision tailored to our research objectives rather than a universally established bibliometric standard. While this targeted temporal focus successfully captures the current dynamic phase, it inherently means that studies from earlier exploratory phases were excluded. Consequently, our findings primarily reflect the developmental trajectory of the field over the past decade, offering limited representation of its complete historical evolution.

On a procedural note, the necessary preprocessing step of manual data standardization to resolve inconsistencies in author and institutional names inherently introduces a minor risk of subjective interpretation. To comprehensively advance this domain, future investigations should incorporate a broader range of databases alongside exhaustive systematic qualitative reviews to seamlessly validate and complement these macroscopic findings.

## 5 Conclusion

This bibliometric analysis successfully fulfills its designated research objectives by clarifying the developmental trajectory and future directions of electrical stimulation therapies for movement disorders spanning 2016 to 2025. First, in delineating temporal trends and collaborative networks, this study reveals a robust logarithmic growth in global publication output. While the United States and European nations, including England and the Netherlands, drive extensive international collaborations characterized by high network influence, China exhibits substantial publication productivity yet remains structurally isolated with low network centrality. Overcoming these specific localized research silos is imperative for standardizing future multinational clinical trials. Second, our identification of the core knowledge base demonstrates that top-tier multidisciplinary journals such as Movement Disorders and highly cited foundational literature provide the essential methodological frameworks unifying this field.

Third, in tracing the evolutionary trajectory of research hotspots, the findings confirm a definitive scientific transition. The discipline has evolved from its initial focus on validating the safety and clinical efficacy of invasive neuromodulation techniques for classic conditions like Parkinson’s disease toward distinct emerging frontiers. These contemporary frontiers are explicitly defined by mechanistic investigations into neural plasticity, the development of adaptive closed-loop systems, and the integration of advanced neuroimaging to personalize therapeutic interventions. Furthermore, the research landscape is actively expanding to include the diversified application of noninvasive technologies aimed at driving genuine functional recovery.

Ultimately, the current developmental landscape reflects a profound clinical maturation within this domain. The therapeutic paradigm has definitively shifted from traditional symptom suppression toward proactive and dynamically adaptive neuromodulation. To propel this discipline forward, future investigations must prioritize multinational consortia to effectively translate contemporary connectomic discoveries into highly personalized evidence-based treatment protocols. By actively harnessing targeted neuroplasticity rather than merely disrupting pathological neural activity, these advanced stimulation strategies possess the ultimate potential to achieve sustained functional neurorestoration and profoundly elevate long-term clinical outcomes.
