# Supplementary material for: Research hotspots and trends in electrical stimulation for movement disorders: a bibliometric analysis from 2016 to 2025
Source: Front Aging Neurosci. 2026 Apr 23;18:1793056. doi: 10.3389/fnagi.2026.1793056 (PMC13149449; doi:10.3389/fnagi.2026.1793056)
Supplement: Supplementary file 1 [file Table_1.DOCX]

| year | Publications  (n) | Cumulative Publications  (n) | Cumulative Proportion  (%) | Annual Growth Rate  (%) |
| --- | --- | --- | --- | --- |
| 2004 | 232 | 0 | 0 | 0 |
| 2005 | 207 | 439 | 4.501179124 | -10.77586207 |
| 2006 | 292 | 731 | 7.495129704 | 41.06280193 |
| 2007 | 329 | 1060 | 10.86845073 | 12.67123288 |
| 2008 | 373 | 1433 | 14.692915 | 13.37386018 |
| 2009 | 361 | 1794 | 18.3943402 | -3.217158177 |
| 2010 | 382 | 2176 | 22.31108377 | 5.817174515 |
| 2011 | 364 | 2540 | 26.04326874 | -4.712041885 |
| 2012 | 354 | 2894 | 29.67292115 | -2.747252747 |
| 2013 | 425 | 3319 | 34.0305547 | 20.05649718 |
| 2014 | 429 | 3748 | 38.42920127 | 0.941176471 |
| 2015 | 437 | 4185 | 42.90987388 | 1.864801865 |
| 2016 | 482 | 4667 | 47.85194299 | 10.29748284 |
| 2017 | 537 | 5204 | 53.35794115 | 11.41078838 |
| 2018 | 596 | 5800 | 59.46888137 | 10.98696462 |
| 2019 | 541 | 6341 | 65.01589255 | -9.228187919 |
| 2020 | 540 | 6881 | 70.55265047 | -0.184842884 |
| 2021 | 566 | 7447 | 76.35599303 | 4.814814815 |
| 2022 | 541 | 7988 | 81.9030042 | -4.416961131 |
| 2023 | 536 | 8524 | 87.3987491 | -0.924214418 |
| 2024 | 617 | 9141 | 93.72500769 | 15.1119403 |
| 2025 | 612 | 9753 | 100 | -0.810372771 |

**Supplementary Table S1** Annual publication statistics and growth rates for electrical stimulation in movement disorders from 2004 to 2025
